# Patient-specific 3D-printed shelf implant for the treatment of hip dysplasia tested in an experimental animal pilot in canines

**DOI:** 10.1038/s41598-022-06989-9

**Published:** 2022-02-22

**Authors:** Koen Willemsen, Marianna A. Tryfonidou, Ralph J. B. Sakkers, René M. Castelein, Martijn Beukers, Peter R. Seevinck, Harrie Weinans, Bart C. H. van der Wal, Björn P. Meij

**Affiliations:** 1 Department of Orthopedics, University Medical Center Utrecht, HP: 05-228, Heidelberglaan 100, 3584 CX Utrecht, The Netherlands; 2 Department of Clinical Sciences, Faculty of Veterinary Medicine, Utrecht University, Utrecht, The Netherlands; 3 Department of Biomechanical Engineering, Delft University of Technology, Delft, The Netherlands; 4 Department of Radiology, University Medical Center Utrecht, Utrecht, The Netherlands; 5 3D Lab, Division of Surgical Specialties, University Medical Center Utrecht, Utrecht, The Netherlands

**Keywords:** Regenerative medicine, Biomedical engineering, Preclinical research, Musculoskeletal system

## Abstract

The concept of a novel patient-specific 3D-printed shelf implant should be evaluated in a relevant large animal model with hip dysplasia. Therefore, three dogs with radiographic bilateral hip dysplasia and a positive subluxation test underwent unilateral acetabular augmentation with a 3D-printed dog-specific titanium implant. The contralateral side served as control. The implants were designed on CT-based pelvic bone segmentations and extended the dysplastic acetabular rim to increase the weight bearing surface without impairing the range of motion. Outcome was assessed by clinical observation, manual subluxation testing, radiography, CT, and gait analysis from 6 weeks preoperatively until termination at 26 weeks postoperatively. Thereafter, all hip joints underwent histopathological examination. The implantation and recovery from surgery was uneventful. Clinical subluxation tests at the intervention side became negative. Imaging showed medialization of the femoral head at the intervention side and the mean (range) CE-angle increased from 94° (84°–99°) preoperative to 119° (117°–120°) postoperative. Gait analysis parameters returned to pre-operative levels after an average follow-up of 6 weeks. Histology showed a thickened synovial capsule between the implant and the femoral head without any evidence of additional damage to the articular cartilage compared to the control side. The surgical implantation of the 3D shelf was safe and feasible. The patient-specific 3D-printed shelf implants restored the femoral head coverage and stability of dysplastic hips without complications. The presented approach holds promise to treat residual hip dysplasia justifying future veterinary clinical trials to establish clinical effectiveness in a larger cohort to prepare for translation to human clinic.

## Introduction

Hip dysplasia or developmental dysplasia of the hip affects as many as one in every 22 newborns^1^. However, cases that eventually need treatment have an incidence of 0.5%^2,3^. Over the last decades, treatment outcome has improved by treating young patients before their triradiate cartilage definitely closes at the age of approximately 16 years^4^. However, early detection and treatment sometimes fails, leading to (young) adolescents with residual dysplasia who present with pain or pre-osteoarthritic changes^5^. It is presumed that many osteoarthritic hips are the result of (subclinical) dysplastic hips^6,7^. In skeletal mature cases of hip dysplasia, surgical treatment is often indicated to prevent severe osteoarthritis at later age^6^.

The gold standard surgical treatment option for hip dysplasia is Peri-Acetabular Osteotomy (PAO)^8^. PAO is an invasive surgery with an extensive learning curve and is associated with a high rate of complications^8^. Therefore, the concept of shelf arthroplasty^9^ could be revisited by using titanium additive manufacturing technologies^10^ to develop 3D-printed joint preserving implant in a personalized approach^11,12^.

The titanium 3D-printed shelf implant was previously biomechanically tested in a cadaveric dog model and demonstrated to stabilize the dysplastic hip joint by creating an acetabular rim extension in a predictable and consistent manner^11^. Similar to the autologous shelf arthroplasty the 3D shelf implant is placed extra capsular with the synovial membrane lining the inner rim of the implant and thereby increasing the weight bearing surface of the dysplastic acetabulum.

An experimental animal model should be used to investigate the concept and feasibility of the 3D-printed shelf implant. Dogs are the animal of choice for a translational study as canine hip dysplasia has similar diagnostic and treatment strategies as developmental hip dysplasia in humans^13,14^. The primary aim of the study is to test the feasibility and safety of the 3D shelf implantation in a small pilot of three experimental dogs, because when implantation is proven safe an immediate translation is preferred to symptomatic patient dogs who consult the veterinarian. As secondary outcomes the post-operative rehabilitation of the implantation is followed and compared to the control side using clinical observation, manual subluxation testing, imaging, gait analysis and post mortem histology of the hip joint.

## Materials and methods

### Ethics approval

Animal handling was in accordance with the European Directive for the Protection of Vertebrate Animals Used for Experimental and Other Scientific Purposes (86/609/EU). The experiments were approved by the National Central Committee for Experiments on Animals (CCD) and a maximum of three experimental dogs could be used to evaluate safety (AVD1080020173505) after which a new clinical trial should be started to investigate effectiveness in symptomatic dog patients. The working protocol (WP3505-01-1) was further supervised by the local Animal Welfare Body and followed the ARRIVE guidelines.

### Study design

Prior to the implantation of the personalized 3D-printed implant (T = 0) and during the 6 months follow up period, clinical observation, manual subluxation testing, imaging, gait analysis were conducted. Upon termination of the study, histology of the hip joints was performed (Table 1).

**Table 1:**
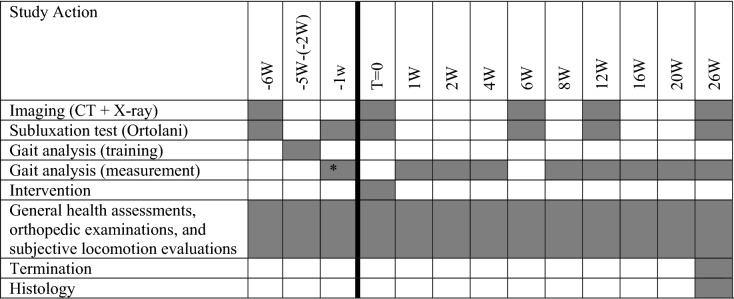
Study outline in weeks (W). T = 0 is the time point of intervention.

*The preoperative baseline consisted of three measurements conducted on separate days during 1 week.

### Animals

**Figure 1:**
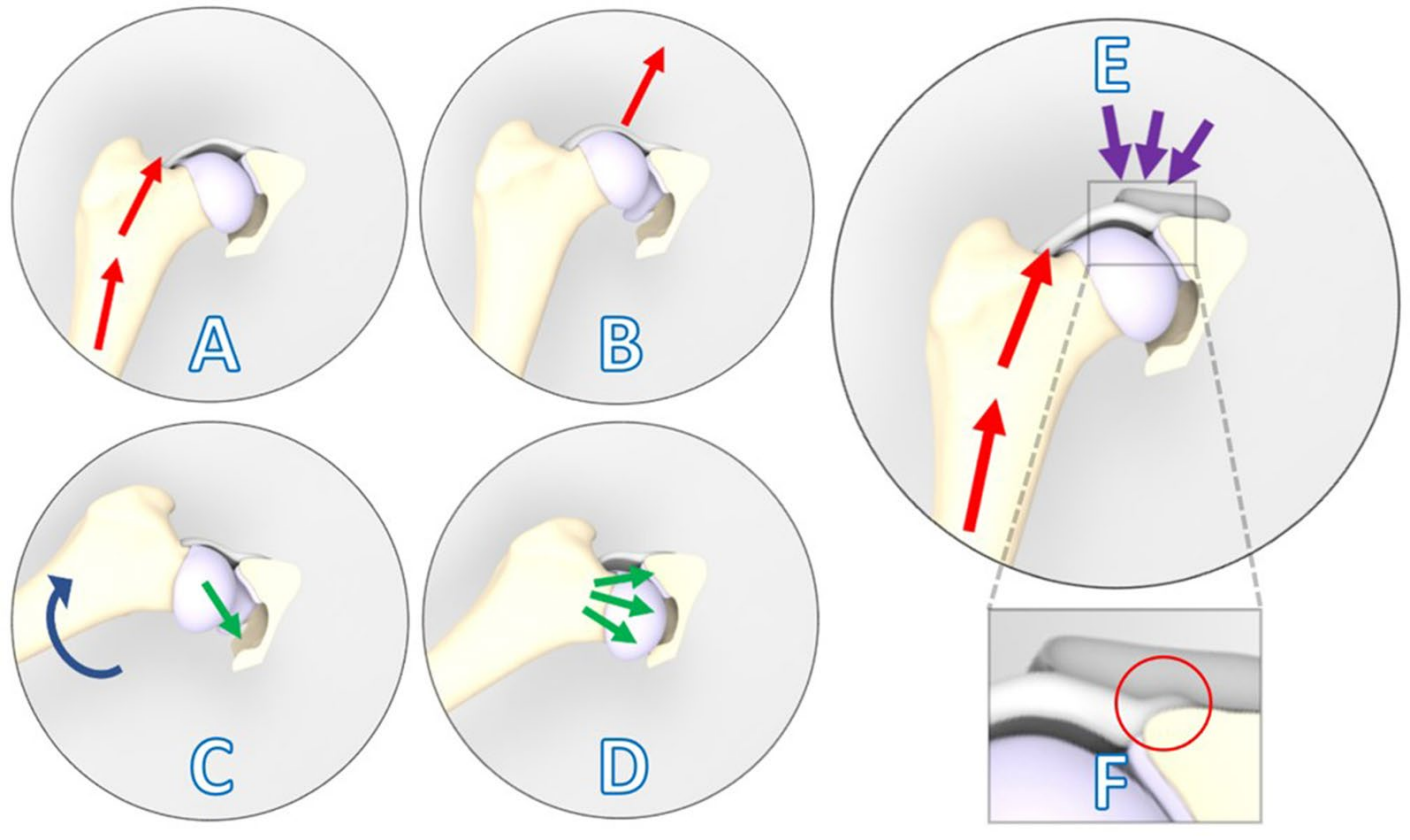
Laxity due to hip dysplasia is confirmed based on clinical examination (A-D) and is counteracted by implantation of the 3D-printed shelf implant (E). (**A**) The limb is in neutral flexion and in an adducted position, and force is applied toward the dorsum of the dog along the femoral axis (red arrows). (**B**) This force causes dorsal subluxation in a hip with joint laxity due to hip dysplasia. (**C**) During the Ortolani (reduction) test, the limb is slowly abducted (blue arrow) while force on the femur (red arrows) is maintained. (**D**) A positive Ortolani sign is evident when a click is heard or palpated as the subluxated femoral head reduces into the acetabulum (green arrows)^17^. (**E**) Introduction of the shelf implant ideally stabilizes the joint by reinforcing the hip capsule and labrum as a weight bearing and stabilizing surface (purple arrows). In close-up the internal 2 mm offset of the implant is visible that allows the capsule attachment to remain unaffected (**F**).

In this pilot study three female mongrel dogs (Marshall, North Rose, New York) with natural occurring, radiographically confirmed, asymptomatic bilateral hip dysplasia were included. The mean (range) age of the dogs was 25 (24–25) months and the mean body weight was 26 (24–29) kg. The hip with the worst dysplastic parameters^15^ based on radiological examination and manual subluxation (Ortolani^16^) testing (Fig. 1A–D) was chosen as the intervention side for the 3D shelf implant (N = 3) and the contralateral hip served as control (N = 3) (Table 2). All subluxation tests were performed under general anesthesia by two board-certified veterinary surgeons who were blinded for each other’s results. The three dogs were housed in a group enclosure with cage enrichment and were put on an ad libitum diet. Furthermore, the dogs were housed with a regular 24-h day-night rhythm and were allowed in an outdoor pen at least twice daily.

**Table 2:** Baseline and postoperative measurements (radiology and Ortolani test).

Measurements	Dog #1		Dog #2		Dog #3	
Hip (L/R)	Left	Right	Left	Right	Left	Right
Operative side	Control	Intervention	Control	Intervention	Intervention	Control
Sex (F/M)	F		F		F	
Weight (kg)	23.9		29.2		23.6	
Age (months)	24		25		25	
**Baseline (**−** 6 weeks)**						
Ortolani	+	+	+	+	+	+
CE-angle (°) 12:00 o’clock	105	98	92	84	99	107
Femoral coverage	53%	48%	46%	41%	50%	53%
Radiographic hip joint incongruency	Mild	Moderate	Moderate and sub-luxation	Moderate and sub-luxation	Moderate	Very mild
**Direct post-operative (+ 0 days)**						
CE-angle (°)	105	120	92	117	120	107
Femoral coverage	53%	61%	45%	59%	61%	53%
Ortolani	+	−	+	+	−	+
Surgical accuracy	–	1 mm	–	3 mm	–	1 mm
**Intermediate follow-up (+ 6 weeks)**						
Ortolani	+	−	+	+	−	−
**Intermediate follow-up (+ 6 weeks)**						
Ortolani	+	−	+	−	−	+
**Final follow-up (+ 6 months)**						
CE-angle (°)	104	120	92	116	119	108
Femoral coverage	52%	62%	46%	58%	60%	54%
Ortolani	+	−	−	−	−	+

*L* left, *R* right, *F* female, *M* male, *CE* center edge angle.

### The intervention/imaging

At the initiation of the study (− 6 weeks), a CT-scan with a standardized protocol (Appendix 1) was made of the entire pelvic area and femora (120 kV, 250 mas, 0.6 mm slice thickness). The CT scans were semi-automatically segmented using imaging processing software, Mimics Medical 21.0 (Materialise, Leuven, Belgium). Standardized bone threshold values (HU 226—upper boundary) were used to guide the semi-automatic CT-based anatomical model. This model was saved and transferred using Stereolithography (STL file) to design the 3D shelf implant.

### Implant design

**Figure 2:**
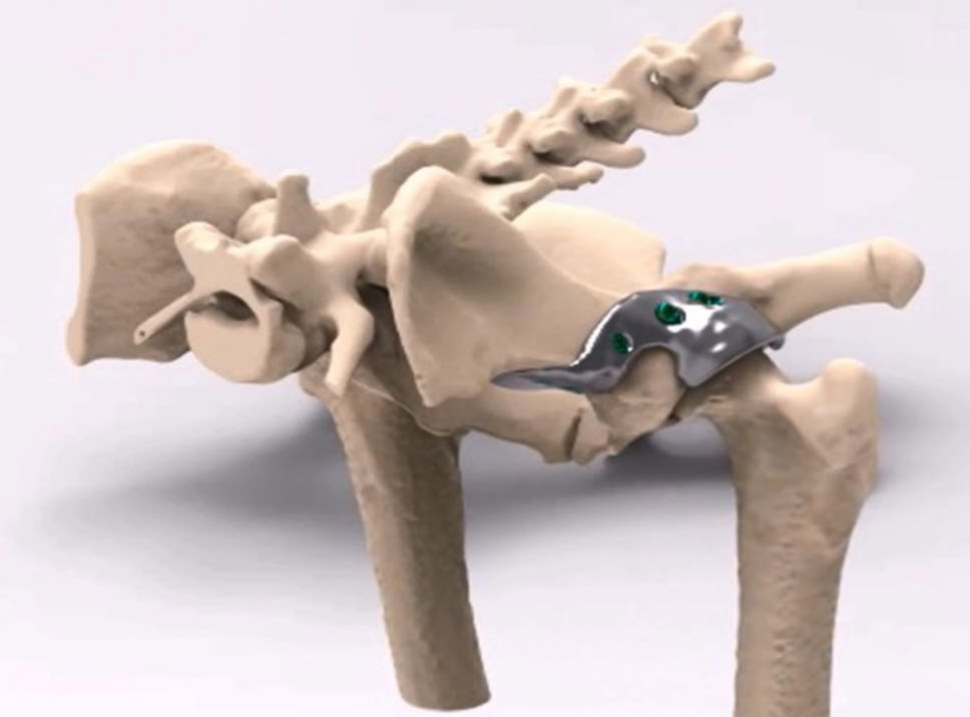
Rendering of a canine pelvis with a 3D-designed acetabular rim implant for the left dysplastic hip. Orientation: left is cranial, top is dorsal.

**Figure 3:**
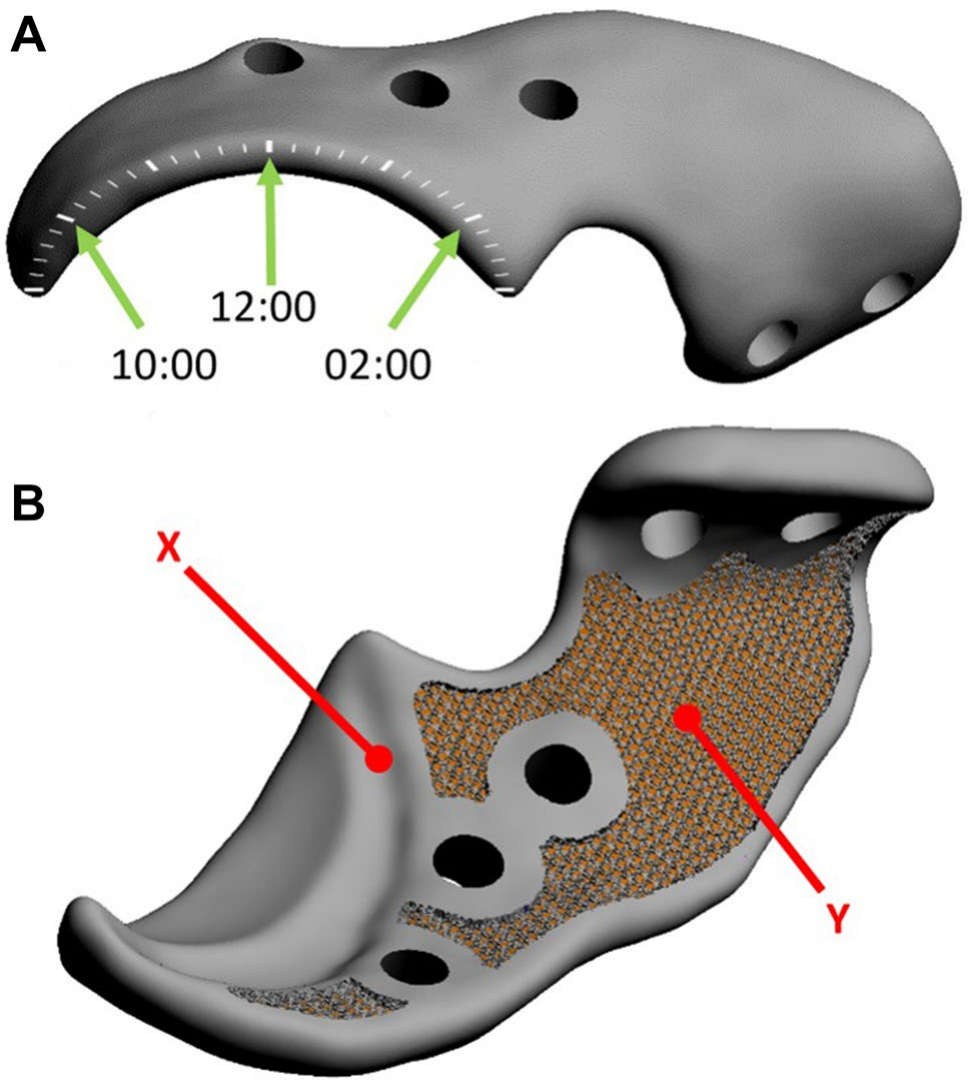
Digital rendering of the implant designed for dog #1. (**A**) The external implant surface with the clockface positions (green arrows) on the rendered implant. (**B**) The internal implant surface shows the internal offset (*X*) that allows the capsule attachment to remain unaffected and the 70% porous inner shell (*Y*) allowing bone ingrowth for osseous integration and secondary implant fixation.

The patient-specific 3D-printed acetabular shelf implants were designed, by the primary author using Freeform Plus software (Geomagic, 3D Systems, Leuven, Belgium), as described prior by Willemsen et al.^11^ (Figs. 2 and 3). The implants consisted of two subsections; the ‘rim extension part’ and the ‘implant-bone interface or attachment part’. For the rim extension part a 20°–30° increase in CE-angle was pursued and the effect on the range of motion was monitored by performing an in silico range of motion (ROM) simulation, for each individual hip. Thereafter, the outcomes were reviewed with a board-certified surgeon and the design was altered if clinically needed (Figs. 1E, 3) (Video 1). The rim of the acetabulum received an offset of 2 mm not to interfere with the attachment of the joint capsule on the acetabular rim and to allow the hip capsule to be interposed between the implant and the cartilage of the femoral head (Figs. 1F, 3).

The implant-bone interface part was also designed patient specific to be able to incorporate 5 locking screws and an additional ilium flange for ease in positioning and for additional stability. Thereafter, the implant bone interface was designed with a porous (70%, 1 mm sized Dode-Medium unit cell) inner shell to optimize bone ingrowth, osseointegration and secondary implant fixation (Fig. 3). Locking screw holes were planned in the implant in such a way that the screw trajectory remained sufficiently distant from the acetabulum but at the same time purchasing the maximal possible bone stock for the preferred screw length. The screws were placed bi-cortical and generally not parallel to each other.

The implants were manufactured from medical grade titanium alloy Ti-6Al-4V ELI grade 23 by direct metal printing using a ProX DMP320 machine (3D Systems, Leuven, Belgium). Postprocessing included hot-isostatic-pressing, polishing, screw wiretapping and a standard intermediate cleaning step (incl. ultrasonic cleaning and automated cleaning) by the implant manufacturer. Additionally, final (manual) cleaning and autoclave sterilization was performed by our in-house sterilization facility.

Orthogonal radiographs and CT of the pelvis and hips were made at − 6, 0, + 6, + 12, + 26 weeks from the implantation (Table 1) and parameters such as the center-edge (CE)-angle^18^ were assessed by a board-certified veterinary radiologist. Subsequently, the CT-scans were uploaded into image analysis software Mimics (Medical v20, Materialise, Leuven, Belgium) to calculate the percentage of femoral head coverage by using multiplanar reconstruction^19^. The acetabular coverage was measured in + 20° posterior pelvic tilt in relation to the cranial–caudal axis to simulate the functional standing posture of a dog^19^. Additionally, the accuracy of the placement was analyzed by rigidly overlaying the preplanning with the postoperative 3D models with an iterative closest point (ICP) algorithm^20^ and subsequently calculating the average implant transformation matrix in mm^21^.

The surgeries were performed by a board-certified veterinary surgeon under a standardized general anesthesia protocol (Appendix 2b) and consisted of a cranio-dorsal approach (Appendix 3) to the hip joint leaving the joint capsule intact^22^. The implant was fitted to the bone and positioned over the hip joint capsule and was fixated with five 3.5 mm locking screws (DePuys Synthes, Raynham, Massachusetts, USA). Full weight bearing was allowed directly postoperatively and reintroduction of the dog into the study group from an individual cage was done 24 h postoperatively. Due to the surgical nature of the intervention only blinding occurred during histological examination.

### Outcomes

General health assessments, orthopedic examinations, and subjective locomotion evaluations were performed weekly during the whole experiment. The subluxation (Ortolani) tests of the hips were assessed under general anesthesia at − 6, 0, 6, 12, 26 weeks (Fig. 1) (Table 1).

Gait analysis was performed using a standardized gait protocol (Appendix 4) using a force plate^23,24^ for objective evaluation of vertical (Fz) ground reaction forces (N/kg) measuring differences between the intervention and control limb and the distribution ratio between front-limb and hind-limb loading before surgery at − 1 (baseline) and after surgical intervention at 1, 2, 4, 8, 12, 16, 20, and 26 weeks (Table 1).

The dogs were followed for 26 weeks to allow enough time for initial surgical recovery, secondary implant fixation and to asses tissues changes to the joint capsule or cartilage after implant intervention. At final follow‐up, the dogs were euthanized (Appendix 2c). Each hip joint was harvested and macroscopically evaluated before histological examination was performed on the capsule and femoral and acetabular cartilage of the decalcified joints using a standardized staining protocol for Hematoxylin and Eosin (HE) staining, and Safranin O/Fast Green staining^19^ (Appendix 5).

## Results

### Preoperative

**Figure 4:**
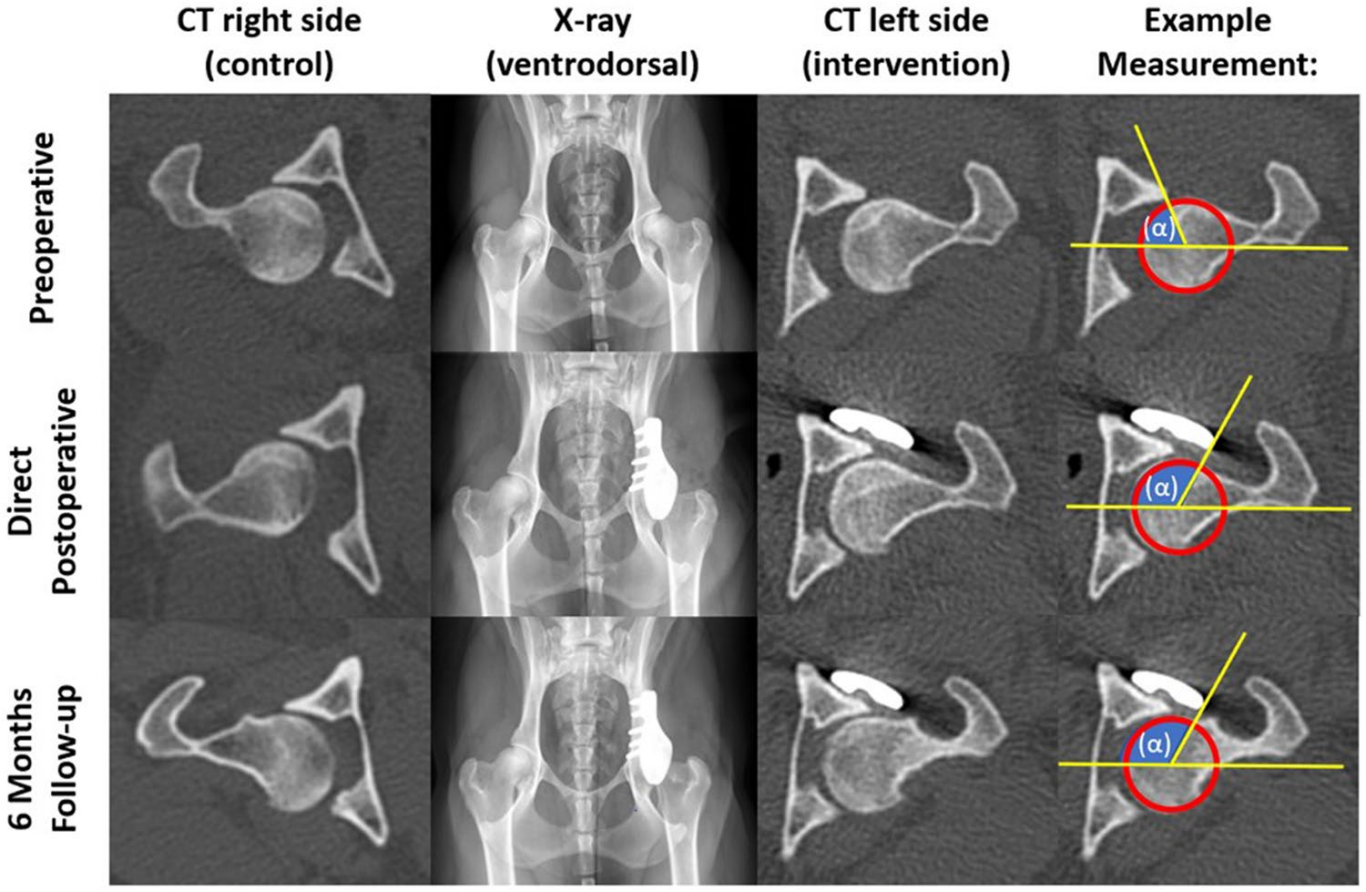
Imaging of dog #3. Preoperatively the intervention side is decentralized. The intervention hip becomes centralized directly postoperatively. On 6 months follow-up the intervention side is still centralized showing improvement in comparison to the preoperative situation. The control side remains unchanged. In the right column the change in center of edge (CE) angle (α) is measured on CT. On the postoperative images the head of the femur centralizes in the acetabulum and there is increased dorsolateral coverage of the femoral head which is reflected in an increased CE-angle (α) by measuring the combined rim of the native acetabulum and the rim extension implant. Also it should be mentioned that some osteophytes are visible in the femoral neck at 6 months follow-up, however these were not evidently more present at the control of intervention side.

**Figure 5:**
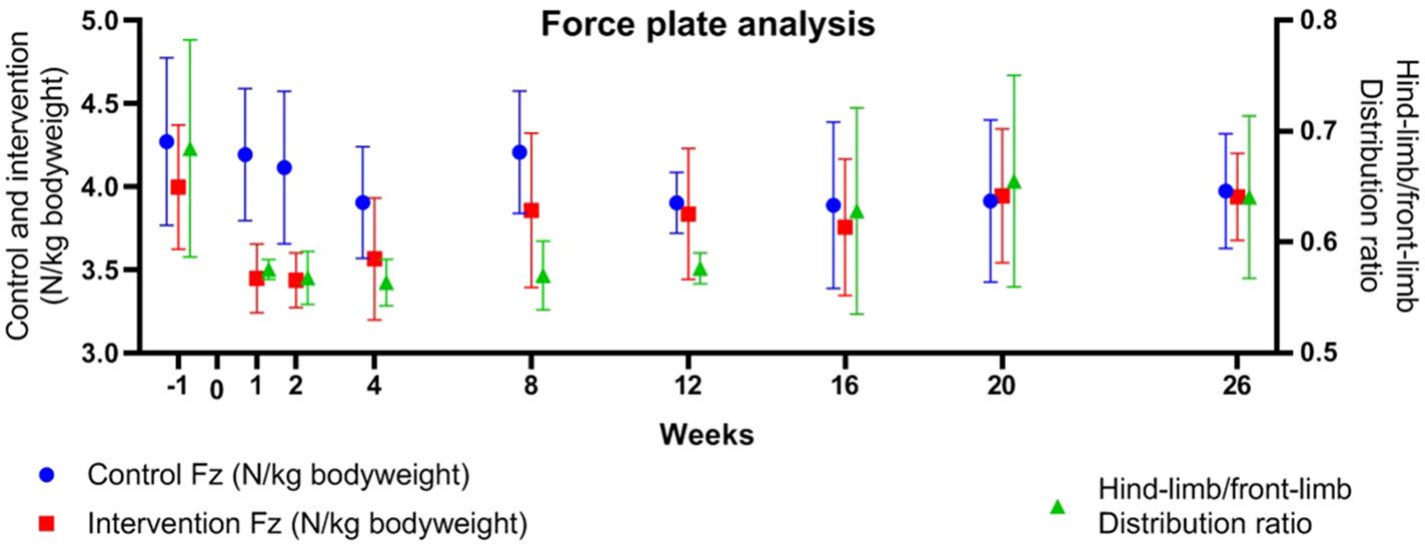
Objective gait analysis by force plate. Mean ± standard deviation force (Fz) (N/kg body weight) on the control (red) and intervention (blue) side, and hind-limb/front-limb distribution ratio (green) before and after acetabular rim extension with a personalized 3D implant that was implanted (week 0) in 3 dogs with hip dysplasia.

At baseline (6 weeks preoperative) all intervention and control hips exhibited a positive Ortolani test (Video 2) and femoral heads showed decreased acetabular coverage or subluxation on radiography. The mean CE-angle was 94° (range 84°–99°) for the intervention hips and 98° (range 92°–105°) for the control hips (Table 2; Fig. 4). General orthopedic examination and subjective locomotion evaluation revealed no other relevant joint abnormalities other than the findings related to hip dysplasia. Objective gait analysis showed no marked differences between the loading (Fz) of intervention and control hips (Fig. 5).

### Postoperative

The implantation of the 3D shelf implant went uneventful. Two implants (dog #1 and dog #3) were placed within 1 mm of their planned position while the other implant (dog#2) was placed distally with a 3 mm offset. Directly post-operatively dog #1 and #3 displayed a negative Ortolani test at the intervention side (Video 3). The recovery of all animals was rapid, the dogs were fully weightbearing on the intervention limb the next day. The dogs were comfortable and were able to resume their normal daily activity. At final follow-up, all three intervention hips and one control hip (dog #2) displayed a negative Ortolani test (Table 2) and no screw failure or loosening was witnessed.

After surgery, the mean CE-angle of the treated hips improved, due to a combination of femoral head medialization and an increase in femoral head coverage by the acetabulum and implant (Fig. 4). The mean intervention side CE-angle increased to 119° (117°–120°), which is within the normal range^18^. The mean total femoral head coverage for the intervention hips increased from 46% (range 41–50%) preoperatively to 60% (58–62%) postoperatively (Table 2 and Fig. 4). The radiographic measurements on the control hips did not change over time (Table 2).

During gait analysis in the first two weeks postoperatively, all dogs showed a decrease in their intervention/control ratio of the ground reaction forces. After a mean of four weeks, the intervention/control ratios returned to preoperative levels (Fig. 5). Furthermore, all three dogs showed a decrease of the front limb/hind limb ratio of the ground reaction forces after surgery that returned to baseline levels after an average of 6 weeks (range 3–12 weeks) postoperatively (Fig. 5).

### Histopathology

**Figure 6:**
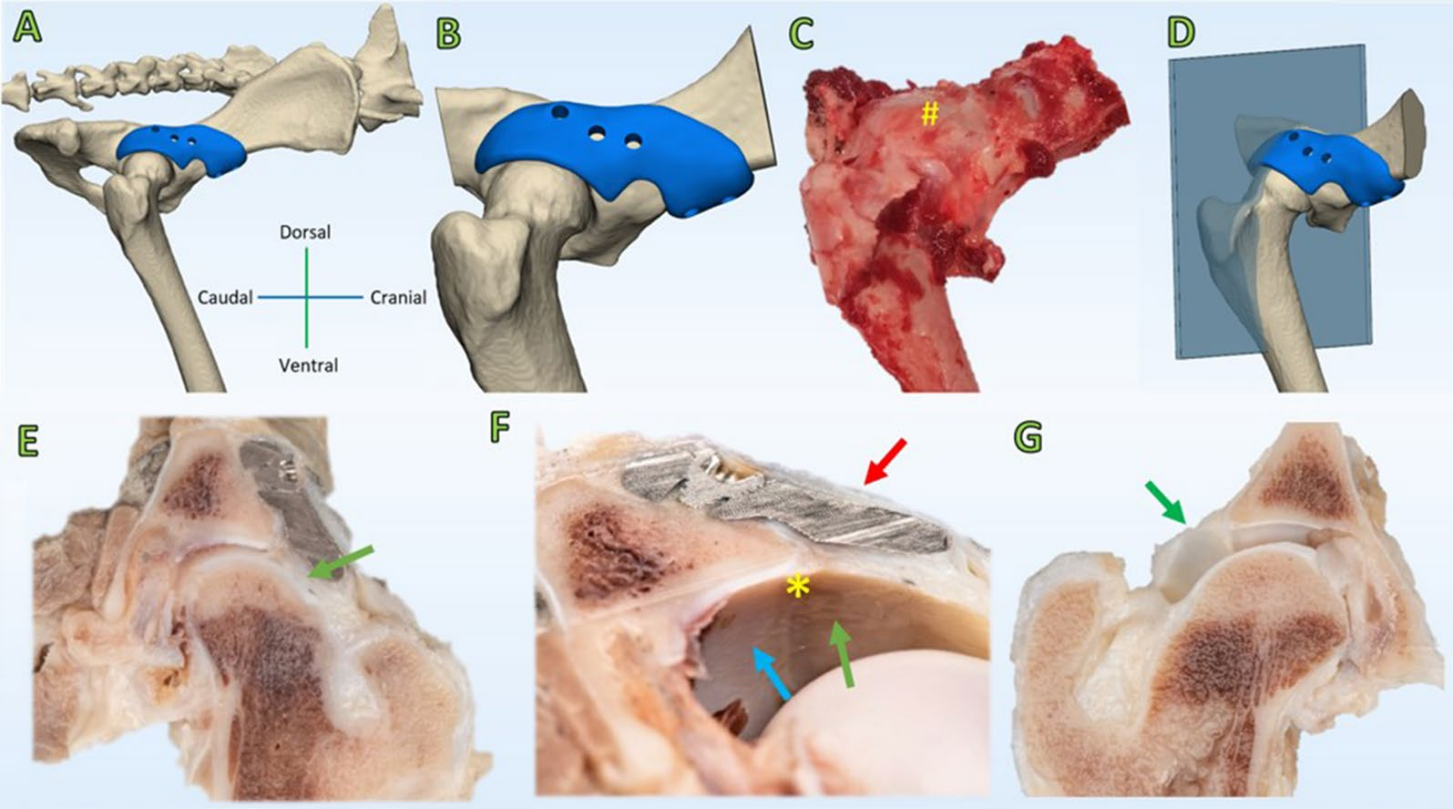
Macroscopy of the intervention and control hip joint of dog#1. (**A**) CT rendering overview of the pelvis with a view on the intervention side. (**B**) CT rendering overview of the size of the dissected specimen. (**C**) Overview of the dissected hip joint with the implant in situ (#). The implant is not distinguishable because it is entirely encapsulated by a thin layer of connective tissue (red arrow, **F)**. (**D**) CT rendering overview of the cut hip plane (Blue) through the 12.00 o’clock position of the acetabulum. (**E**,**F**) Cross section through the 12.00 o’clock position of the acetabulum. The joint capsule shows hypertrophy (green arrow, **E**) and has incorporated the implant in-between layers (red arrow, F) allowing for a smooth transition (*, **F**) from the acetabular cartilage (blue arrow, **F**) into the weightbearing hip capsule (green arrow, **F**). (**G**) The control hip is depicted for reference in a cross section through the 12.00 o’clock position of the acetabulum. The hip capsule has a minimal thickness (green arrow, **G**) as compared to the intervention side (green arrows, **E**,**F**).

**Figure 7:**
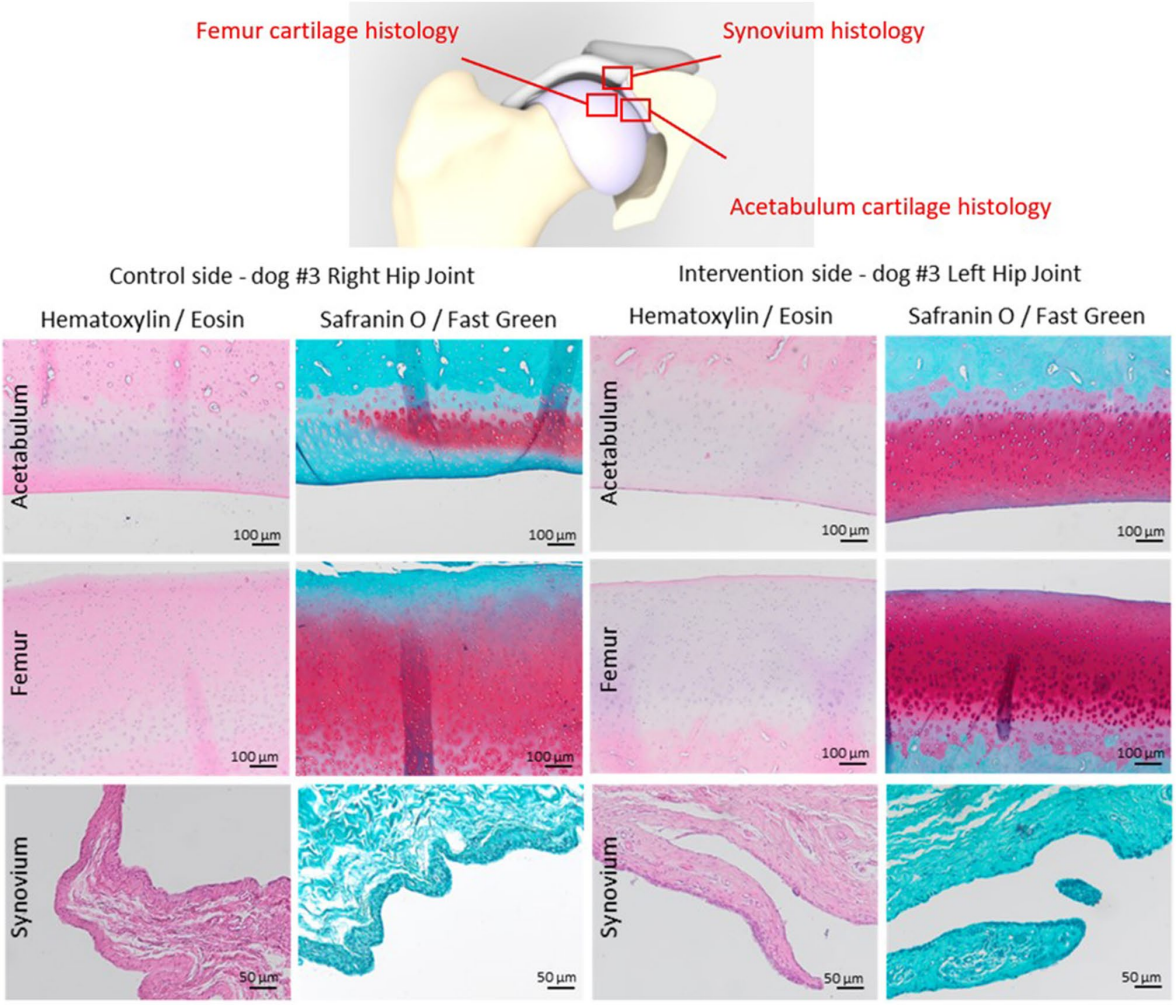
Representative histological images of the acetabular and femoral cartilage and synovium of dog #3. The control side demonstrates a fairly normal volume and smooth surface of acetabular and femoral cartilage, focal loss of proteoglycan staining into the deep zone of the acetabular cartilage, and global loss of proteoglycan staining into the upper zone of the femoral cartilage. The intervention side demonstrates a normal volume and smooth surface of acetabular and femoral cartilage, and unremarkable corresponding Safranin O/Fast Green staining. For both sides there are no abnormalities observed in the tide mark, nor subchondral changes. The synovial lining is composed of 2–3 layers of cells at the control side, whereas at the interventional side it is composed of 1–2 layers of cells. Both sides demonstrate absence of cell infiltrates, and proteoglycan deposition.

Macroscopic evaluation of the hip joints showed that the hip implant was completely encapsulated by connective tissue. When the hips were separated in a cranial and caudal section there was a clear view of the hip capsule at the most dorsolateral (12:00 o’clock) position. The capsule interposed between the implant and the femoral head was markedly increased in thickness compared to control hips, and had a perceptible smooth transition to the macroscopically unaffected acetabular cartilage (Fig. 6). The samples presented variable pathologic changes of cartilage structure, varying from normal volume with smooth cartilage surface with all zones intact (OARSI grade A) to fissures to the mid zone and erosion of the surface (OARSI grade C). No additional histological damage to the acetabular or femoral cartilage, or metallosis due to the implant was observed in the intervention hips compared to the control hips (Fig. 7). Likewise, the synovial membrane presented with variable levels of absent (control hip dog#1 and all intervention hips) to mild synovitis (control hips of dog#2 and dog#3) evidenced by an increase in the number of cell layers (up to 3) and finger-like villous hyperplasia. Full histological results are presented in the Appendix 6.

## Discussion

The present study provides a *proof-of-concept* for a safe and feasible surgical approach to treat naturally occurring hip dysplasia with a personalized 3D-printed titanium shelf implant in a dog model. The shelf implant augmented the acetabular rim and was effective in increasing femoral head coverage and normalizing the CE-angle of the dysplastic hip joint. The in vivo implantation of the 3D shelf implants demonstrated minimal morbidity, uneventful recovery, and normalization of the gait of the dogs to baseline based on force plate analysis while preserving joint health.

The experimental dogs were allowed full weight-bearing immediately after implantation without clinical adverse effects, which confirmed the implant safety derived from the break-out test in the reported biomechanical study with cadaveric dysplastic dog hips^11^. Cartilage health remained preserved based on macroscopic and histological findings 6 months after implantation. Altogether these observations indicate that the 3D shelf implant can be used in a clinical setting to treat dogs suffering from hip dysplasia, and may not require postoperative lifestyle restrictions following a standard period of limited exercise restriction.

Surgical interventions in case of hip dysplasia are primarily meant to improve hip joint stability and preserve joint health^6^. The 3D shelf implant helped to improve the stability of the hip joints as all three intervention hips demonstrated negative Ortolani tests at final follow-up in combination with medialization of the femoral head to a normal position on CT scan images. This was in agreement with a prior biomechanical study which revealed that the 3D shelf implant added stability to the hip joint^25^. While the control hips of two dogs still showed hips with subluxation, one control hip (dog #2) also presented a negative Ortolani at 6 months postoperatively, albeit with evident signs of osteoarthritic changes on histology. This is a well-known phenomenon, as negative Ortolani testing is commonly seen in dogs with hip dysplasia that develop progressive secondary osteoarthritis commencing from their second year of adult life^26^. In future studies fluoroscopy examination under sedation and weight bearing conditions^27^ or during treadmill walking^28^ could be used to give more insight in hip joint stability during follow up of the 3D shelf implant.

Improved joint stability within 6 months after implantation of the 3D shelf implant is most probably achieved by a combination of increased femoral head coverage and soft tissue changes after implantation. In all three dogs, the capsule lining the inside of the titanium acetabular rim completely filled the (2 mm) gap between implant and femoral head and was remarkably thicker than the submillimeter natural thickness of the normal hip capsule^29^. This suggests that the increased biomechanical requirements of the capsule resulted in hyperplasia of the synovial membrane without inducing synovitis. Also in dog #2, where implant positioning was slightly imperfect, the space between the implant and femoral head was macroscopically filled with a relatively thick tissue layer. The latter probably contributed to the stabilization of the hip joint and resulted in a negative Ortolani test and facilitated the return to baseline locomotion in similar fashion as in the other two experimental hips. A further favoring of the treated (hind) limb was not anticipated as these experimental dogs were not (yet) clinically affected by their dysplasia and therefore a conversion to a clinically affected patient population is essential.

The 3D shelf implant as a treatment resembles the shelf arthroplasty that has been described in dogs using a biocompatible osteoconductive polymer (BOP)^30^. Although short-term clinical effectiveness of the BOP shelf arthroplasty was reported in dogs with hip dysplasia, a study in normal dogs showed that ossification around the BOP fibers was slow and unsatisfactory to recommend its use for the treatment of canine hip dysplasia^30^. In another prospective study, 10 dogs with bilateral hip dysplasia were treated with the BOP shelf arthroplasty on the right hip joint and a sham procedure on the left hip joint^31^. Large bony shelfs failed to develop on the treated hips and the amount of periarticular bone even decreased over time. The BOP implants were encapsulated by fibrous tissue and there was no histologic evidence of osteoconduction by the bony implants^31^. Shelf arthroplasty using the 3D printed titanium implant in the present study has the advantage that it is not dependent on osteoconduction or osteoinduction, results in immediate patient-specific augmentation of the acetabular rim, and potentially limits uncontrolled bone proliferation. However, no histology was performed on the porous sections of the implant to review the amount of bone ingrowth and this is still recommended for future research in a clinically affected cohort with longer follow-up.

Hip dysplasia morphology, diagnostics, and treatment options in both man and dogs are comparable allowing for a translational study employing dogs as a model to show a proof-of-concept^13^. Ethical considerations prevented the use of a higher number of experimental dogs. Nonetheless, this study serves the veterinary dog patient suffering from canine hip dysplasia, to offer an alternative for invasive double or triple pelvic osteotomies, or prevent future femoral head and neck resection or hip joint replacement^32^. Long term follow up studies in patient dogs may give insight in whether this procedure may prevent the development of debilitating secondary hip osteoarthritis before evaluating this procedure for humans. Within this context, there are some limitations of the current study. First, the follow-up can be deemed as short and numbers treated small, however if no implant failure was witnessed in the first few months under full weight bearing, the ethical committee had enough confidence to allow a secondary trial in clinically affected canine patients in which the effect of the implant shall be further evaluated. Another limitation of this study with respect to its translation towards humans is the analogous hip anatomy of dogs and humans that exhibits marked functional differences with respect to loading in magnitude, direction and the front/hind limb weight ratio that logically differs between quadruped animals and biped humans^33^. Therefore, to prepare translation of this 3D shelf approach to human clinic a cadaveric proof of concept is required with a further biomechanical analysis.

This study showed a proof-of-concept of a patient-specific acetabular rim implant that restored the coverage and stability of dysplastic hip joints to a normal level without complications. This low invasive procedure holds promise to treat dog patients with hip dysplasia. To compare this novel procedure to the gold-standard TPO and confirm long term safety and efficacy, a follow up study with a larger cohort in a clinically affected dog population suffering from hip dysplasia is indicated. To prepare translation of this 3D shelf approach to human clinic a cadaveric proof of concept is also required.

## Supplementary Information

Supplementary Information.

Supplementary Video 1.

Supplementary Video 2.

Supplementary Video 3.
