# Supplementary material for: Patient-specific 3D-printed shelf implant for the treatment of hip dysplasia tested in an experimental animal pilot in canines
Source: Sci Rep. 2022 Feb 22;12:3032. doi: 10.1038/s41598-022-06989-9 (PMC8863847; doi:10.1038/s41598-022-06989-9)
Supplement: Supplementary file 1 — Supplementary Information. [file 41598_2022_6989_MOESM1_ESM.pdf]

Appendix

Journal Name:

Nature, Scientific Reports

Title:

Patient-specific 3D-printed shelf implant for the treatment of hip dysplasia: an experimental animal pilot in canines

Author names:

Koen Willemsen, MD[1,5,*] (Orcid: 0000-0002-8237-6321)

Marianna A. Tryfonidou, DVM, PhD[2]

Ralph J.B. Sakkers, MD, PhD[1]

René M. Castelein, MD, PhD[1]

Martijn Beukers, DVM[2]

Peter Seevinck, PhD[4]

Harrie Weinans, PhD[1,3]

Shared last author: Bart C.H. van der Wal, MD, PhD[1]; Björn Meij, DVM, PhD[2]

1. Department of Orthopedics, University Medical Center Utrecht, The Netherlands
2. Department of Clinical Sciences, Faculty of Veterinary Medicine, Utrecht University, The Netherlands
3. Department of Biomechanical Engineering, Delft University of Technology, The Netherlands
4. Department of Radiology, University Medical Center Utrecht, The Netherlands
5. 3D lab, Division of Surgical Specialties, University Medical Center Utrecht, The Netherlands

* Corresponding author; e-mail address: k.willemsen-4@umcutrecht.nl, Tel: +31887559001, postal address: K. Willemsen, HP: 05-228, Heidelberglaan 100, 3584 CX Utrecht, The Netherlands

Table of contents:

1. Standardized CT-scan protocol
2. Anesthesia and analgesia protocols
    a. Protocol during imaging
    b. Protocol during implantation
    c. Protocol during termination
3. Surgical Approach
4. Gait analysis
5. Histology (Methods)
6. Histology (Results)
7. Video’s

References

1. Standardized CT-scan protocol

Under a general anesthesia protocol (Appendix 1.5.1) the dogs were positioned in dorsal recumbency with extended and slightly internally rotated femora, consistent with hip dysplasia (HD) position I for radiographic canine HD screening programs (Fig. S1). In the ventrodorsal HD position I radiograph the pelvis is exactly symmetrical, left and right femur are parallel and the patella is positioned midline on the distal femur. The CT scans were made using a 64 slice CT scanner (Siemens Somatom Definition AS, Siemens Healthcare) with the following standardized parameters: 120 kV, 250 mas, 1000 ms tube rotation time, 0.6 mm slice thickness, 0.35 spiral pitch factor, 512 × 512 pixel matrix. Reconstructions were made in transverse and sagittal planes using soft tissue and bone reconstruction kernels and images were reviewed in soft tissue/bone settings (window length 50, width 300, and window length 600, width 3000, respectively).

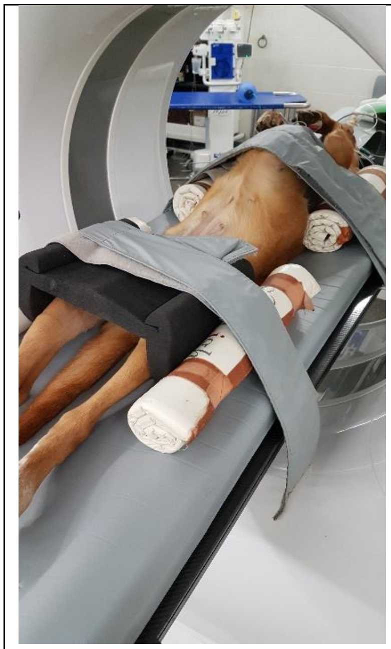

Fig S1.

Standardized position during CT-scan of dog #1 which is similar to ventrodorsal HD position I in routine hip dysplasia screening programs for dogs.

2. Anesthesia and analgesia protocols

a. Protocol during imaging:

For CT and radiographs anesthesia was provided by intravenous dexmedetomidine (10 μg/kg), induction by intravenous propofol (1–2 mg/kg) and maintenance by inhalation of 1–1.5% isoflurane. After imaging, anesthesia was antagonized with atipamezole depending on the administered dose of dexmedetomidine.

b. Protocol during implantation:

For the implantation of the personalized 3D implant anesthesia was provided by preoperative intravenous administration of dexmedetomidine (10 μg/kg), induced by intravenous propofol (1–2 mg/kg) and maintained by intravenous propofol (100 mg/kg/hr) and inhalation of 1–1.5% isoflurane. A morphine injection (0.1 mg/kg) diluted with levobupivacaine (1ml/5kg) was administered in the epidural space between L7 and S1 on top of the cauda equina. After postoperative imaging, the anesthesia was antagonized with atipamezole depending on the administered dose of dexmedetomidine. At induction dogs received antibiotic prophylaxis consisting of cefazoline 20 mg/kg intravenously, repeated every 90 minutes during surgery.

Postoperative analgesia was provided by intravenous buprenorphine for 24 hours (20 μg/kg, one day, three times daily) and subcutaneous carprofen (4 mg/kg). Thereafter analgesia was continued by oral administration of carprofen (2 mg/kg, 7 days, twice a day) and tramadol (Tramagetic once Daily 200 mg, 3-7 days on indication, twice a day. Postoperative antibiotic prophylaxis consisted of oral administration of amoxycillin / clavulanic acid (Synulox; 12.5 mg/kg PO, 7 days, twice a day).

c. Termination:

At T = 6 months, the animals received intravenous pre-medication consisting of dexmedetomidine (10 μg/kg) and butorphanol (0.1 mg/kg) followed by euthanization with intravenous pentobarbital (200 mg/kg).

3. Surgical approach

All implantations were performed by one surgical team (a board certified veterinary surgeon and a medical doctor) to prevent variations in placement and screw insertion torque.

The skin incision was centred at the level of the greater trochanter and was placed over the cranial border of the shaft of the femur. Distally, it extended one third the length of the femur; proximally, it curved slightly cranially to end just short of the dorsal midline. The skin margins were undermined and retracted. An incision was made through the superficial leaf of the fasciae latae, along the cranial border of the biceps femoris muscle. The biceps femoris muscle was retracted caudally to allow incision in the deep leaf of the fasciae latae to free the insertion of the tensor fasciae latae muscle. The incision continued proximally through intermuscular septum between the cranial border of the superficial gluteal muscle and the tensor fasciae latae muscle. The fasciae latae and the attached tensor fasciae latae muscle were retracted cranially and the biceps caudally. Blunt dissection and separation along the neck of the femur with the fingertip allowed visualization of a triangle bounded dorsally by the middle and deep gluteal muscles, laterally by the vastus lateralis muscle, and medially by the rectus femoris muscle. The insertion of the rectus femoris muscle was exposed by tenotomy of a portion of the deep gluteal tendon close to the trochanter, leaving enough tendon on the bone to allow suturing. The deep gluteal muscle was split proximally, parallel to its fibres, and the pedicle was allowed to retract exposing the cranial and dorsal rim of the acetabulum. Using a periosteal elevator, the deep gluteal muscle was elevated from the bone cranially and dorsally to the acetabulum to free the acetabular rim for the acetabular rim extension implant. The joint capsule was covered by areolar tissue, which was carefully cleared away by blunt dissection without opening the joint. The ilium bone was exposed ventrally, just cranial to the rectus femoris muscle to allow the implant to curve around the ventral border of the

ilium. The implant was fitted to its designated site at the rim of the acetabulum and after rechecking its proper placement, a 3.5 mm drill guide was attached to the middle screw hole and the first hole was drilled with the 2.8 mm drill under continuous irrigation with saline solution. Next, the depth was measured using a depth gauge and double-checked with the pre-operative planning. The appropriate length of a 3.5 mm self-tapping DePuy Synthes locking screw (Raynham, MA, USA) was inserted and locked in the implant. This procedure was repeated for the other holes, alternating cranially and caudally. No fluoroscopy was used during this procedure. Following the placement of the implant the range of motion of the hip joint was tested and assessed for possible impingement of the greater trochanter and the implant. Also, the Ortolani test was repeated with the implant *in situ* and correct placement ideally resulted in a negative Ortolani test.

Closure was started by placing two mattress polydioxanone (PDS 2-0) sutures in the deep gluteal tendon incision, and the origin of the vastus lateralis muscle was sutured to the cranial edge of the deep gluteal muscle. Interrupted polydioxanone sutures (PDS 2-0) were placed in the insertion of the tensor fasciae latae muscle distally and continued proximally along the cranial border of the superficial gluteal muscle. The superficial leaf of the fascia lata distally and the gluteal fascia proximally were closed to the cranial border of the biceps femoris with interrupted polydioxanone sutures (PDS 2-0). The subcutaneous tissue was closed in a continuous layer with poliglecaprone 25 (Monocryl 3-0) and the skin was sutured with polyamide (Ethilon 3-0).

4. Gait analysis

All 3 dogs underwent measurements of ground reaction forces by force plate analysis (Kistler type 9261, Kistler Instrumente AG, Winterthur, Switzerland) in the mediolateral (Fx), craniocaudal (Fy), and vertical (Fz) direction and were normalized for body weight. The force plate saved all recordings for further processing on a computer with a sampling rate of 100 Hz[1,2]. The force plate itself was 60 cm wide and 40 cm long, and was mounted flush with the surface of an 11 m long walkway. The middle 5 m of the runway was bordered by a 50 cm-high fence to guide the dogs over the force plate. Before the first official measurement, a 2h training session was performed three times a week during five weeks to familiarize the canines with the force plate. In the week before implantation three gait analyses per dog were performed on separate days to establish the baseline gait. Force plate analyses were performed at -1 (baseline), 1, 2, 4, 8, 12, 16, 20, and 26 weeks from the intervention. A gait analysis consisted of a minimum of 10 unilaterally paired measurements per side and were always obtained by the same observer with an assistant to minimize inter-observer variation. Ratios between vertical forces on hind limbs versus front limbs were calculated and a comparison was made between the intervention limb and the contralateral control.

5. Histology (Methods)

After six months of follow-up, all dogs were euthanized. Each hip joint was harvested using an electric multipurpose saw (Bosch PSA 700, Gerlingen, Germany)(Main manuscript: Figure 6A-C). The joints were fixated in neutral buffered formalin (NBF 4%, 4286, Klinipath) for at least 16 weeks. Subsequently, the samples were axially transected into an anterior (cranial) and posterior (caudal) part through the 12:00 o'clock clockface position using a diamond band saw (EXAKT 312)(Main manuscript: Figure 6D-G). Next, the implants were removed and samples were decalcified during 14 days with the aid of Formical-4 at 37°C maintained by a microwave, embedded in paraffin and sections of 5µm were fixed on Microscope Ultra plus slides (KP-3056, Klinipath). All Hematoxylin and Eosin, Safranin O/Fast Green stained sections of cartilage and synovium were blindly evaluated by two authors employing parameters as proposed by the Osteoarthritis Research Society International (OARSI) for the basic assessment for osteoarthritis for the dog.[3] Cartilage structure, chondrocyte (pathology) and proteoglycan staining were assessed at the level of the load-bearing surface (12:00 o'clock position) of the acetabulum and the femoral head. The synovial membrane was assessed at the dorsal acetabulum-synovium interface underneath the implant in the intervention hips or the equivalent location in the control hip joint.

6. Histology (Results)

Figure S2 Full histological results

Schematic illustration of the locations of described histology samples:

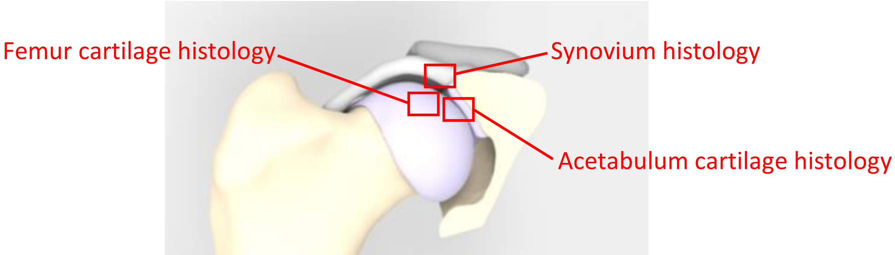

Dog 1: 120512

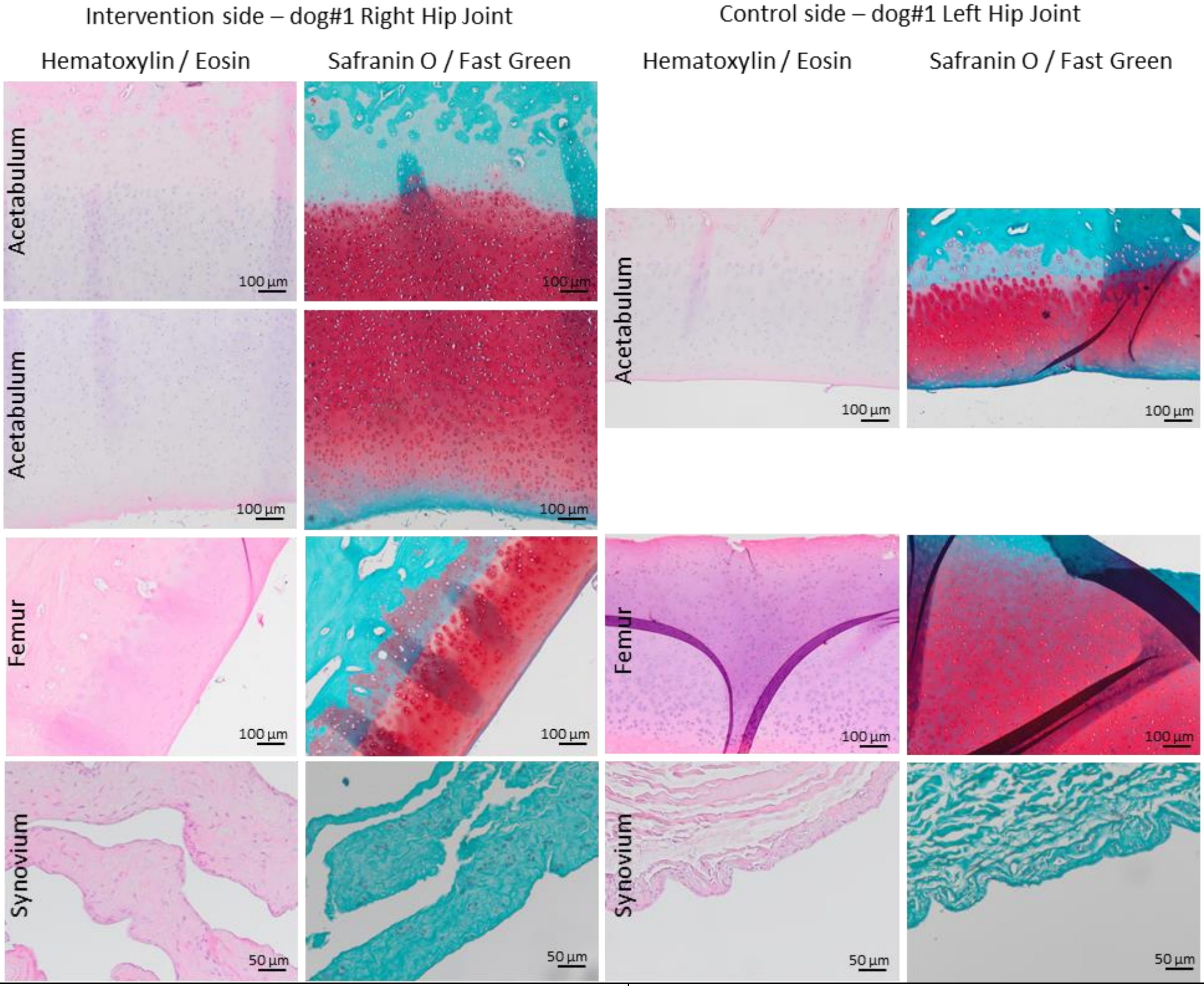

Intervention side (Right)	Control side (Left)
The intervention side demonstrated surface undulation of the acetabular cartilage with loss of cells in the upper zone. Safranin O/Fast Green staining demonstrated loss of proteoglycan content in the upper zone. There were no abnormalities observed in the tide mark, nor subchondral changes. Synovial lining was composed of 1-2 layers of cells,	The control side demonstrated a fairly normal volume of cartilage with smooth surface and all zones of the acetabular and femoral cartilage were intact with focal surface undulation and focal loss of cartilage cells in the upper zone. The corresponding Safranin O/ Fast Green staining appeared to be normal. There were no

while villous hyperplasia and cell infiltrates were absent. In synovium sections stained for safranin O/Fast Green staining, cell clustering was evident.	abnormalities observed in the tide mark, nor subchondral changes. Synovial lining was composed of 1-2 layers of cells, while villous hyperplasia and cell infiltrates and proteoglycan deposits were absent

**Dog 2: 117952**	
**Intervention side (Right)**	**The control side (Left)**
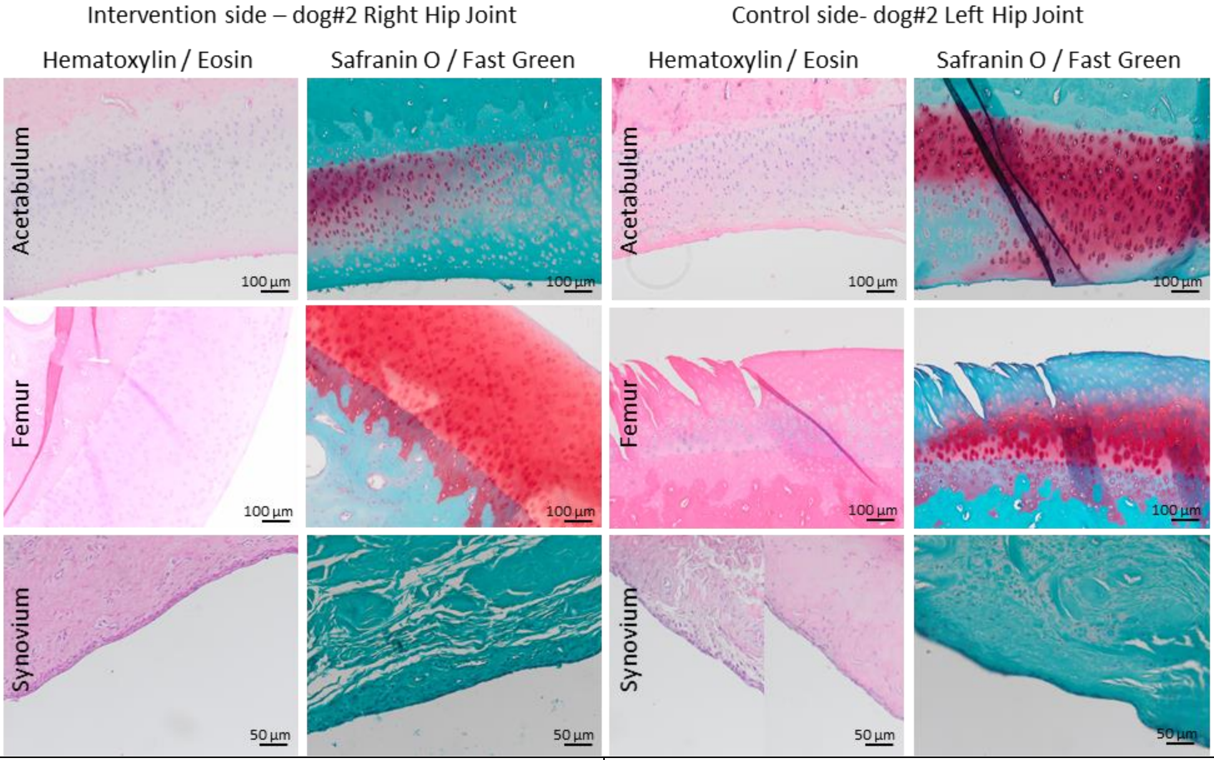	
The intervention side demonstrated mild undulation of the acetabular cartilage with loss of cells in the surface zone and occasionally small cell clusters. Safranin O/Fast Green staining demonstrated decreased proteoglycan content into the mid zone and focally into the deep zone. There were no abnormalities observed in the tide mark, nor subchondral changes. Synovial lining was composed of ~3 layers of cells, while villous hyperplasia and cell infiltrates were absent; proteoglycan deposition was absent.	The control side demonstrated fissures to the mid zone and erosion of the upper zone, with predominantly loss of cells in the surface zone and occasional small cell clusters in the mid zone of the femoral head cartilage. Safranin O/Fast Green staining demonstrated decreased proteoglycan content into the mid zone and focally into the deep zone. Synovial lining was composed of ~3 layers of cells, finger-like, villous hyperplasia and mild to moderate inflammatory cell infiltrates. In the subsynovial layer there was weak safranin O staining indicative of chondrification of the synovial cells.

**Dog #3 (117979)**

The control side (Right)	Intervention side (Left)
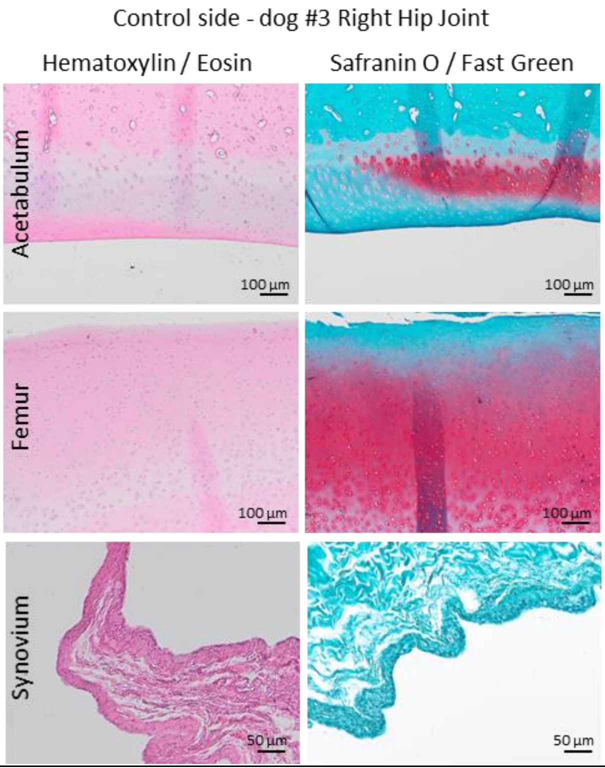	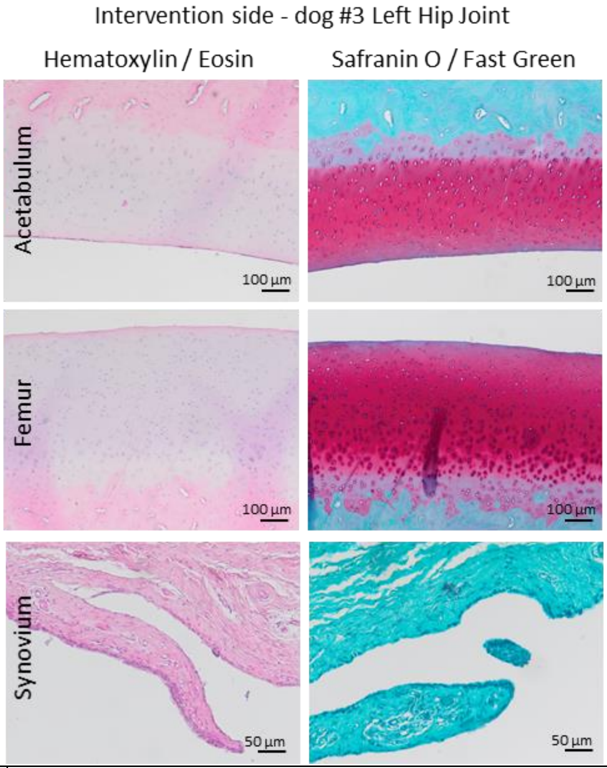
The control side demonstrated a fairly normal volume of cartilage with smooth surface and all zones intact of the acetabular and femoral cartilage with focal loss of proteoglycan staining into the deep zone of the acetabular cartilage and global loss of proteoglycan staining into the upper zone of the femoral cartilage. There were no abnormalities observed in the tide mark, nor subchondral changes. Synovial lining was composed of 2-3 layers of cells, while villous hyperplasia, cell infiltrates, and proteoglycan were absent.	The intervention side demonstrated a normal volume of cartilage with smooth surface and all zones of the acetabular and femoral cartilage were intact. The corresponding Safranin O / Fast Green appeared also to be normal. There were no abnormalities observed in the tide mark, nor subchondral changes. Synovial lining was composed of 1-2 layers of cells, while villous hyperplasia and cell infiltrates were absent. Synovium was negative for proteoglycan and collagen type II.

7. Video's

Video 1.

In silico 3D model of the hind limb of a dog showing hip instability due to a dysplastic acetabular rim followed by the virtual implantation of a 3D printed shelf implant. After implantation the hip stability improves without limiting the hip's range of motion.

Video 2.

Video demonstrating the preoperative Ortolani test in dog #1. A distinct audible and palpable click is observed indicating reduction of the femoral head in the acetabulum marking a positive Ortolani test.

Video 3.

Video demonstrating the postoperative Ortolani test in dog #1. No audible or palpable click is observed indicating a stable hip joint that does not allow subluxation of the femoral head marking a negative Ortolani test.

References:

1. Tellegen, A. R., Willems, N., Tryfonidou, M. A. & Meij, B. P. Pedicle screw-rod fixation: a feasible treatment for dogs with severe degenerative lumbosacral stenosis. *BMC Vet. Res.* **11**, 299 (2015).

2. Suwankong, N. *et al.* Assessment of decompressive surgery in dogs with degenerative lumbosacral stenosis using force plate analysis and questionnaires. *Vet. Surg.* **36**, 423–431 (2007).

3. Larson, C. M. *et al.* Are normal hips being labeled as pathologic? A CT-based method for defining normal acetabular coverage. *Clin. Orthop. Relat. Res.* **473**, 1247–1254 (2015).
